# Effect of Daytime and Tree Canopy Height on Sampling of *Cacopsylla melanoneura*, a ‘*Candidatus* Phytoplasma mali’ Vector

**DOI:** 10.3390/plants9091168

**Published:** 2020-09-09

**Authors:** Dana Barthel, Christine Kerschbamer, Bernd Panassiti, Igor Malenovský, Katrin Janik

**Affiliations:** 1 Laimburg Research Centre, Laimburg 6, Pfatten (Vadena), IT-39040 Auer (Ora), South Tyrol, Italy; christine.kerschbamer@laimburg.it (C.K.); bernd.panassiti@gmail.com (B.P.); 2 Department of Botany and Zoology, Faculty of Science, Masaryk University, Kotlářská 2, 61137 Brno, Czech Republic; malenovsky@sci.muni.cz

**Keywords:** apple proliferation, phytoplasma, insect, vector, *Cacopsylla picta*, Psyllidae, monitoring, beating tray, wash down, nymph, temperature

## Abstract

The psyllids *Cacopsylla melanoneura* and *Cacopsylla picta* reproduce on apple (*Malus × domestica*) and transmit the bacterium ‘*Candidatus* Phytoplasma mali’, the causative agent of apple proliferation. Adult psyllids were collected by the beating-tray method from lower and upper parts of the apple tree canopy in the morning and in the afternoon. There was a trend of catching more emigrant adults of *C.* *melanoneura* in the morning and in the lower part of the canopy. For *C.* *melanoneura* remigrants, no differences were observed. The findings regarding the distribution of adults were reflected by the number of nymphs collected by wash-down sampling. The density of *C.* *picta* was too low for a statistical analysis. The vector monitoring and how it is commonly performed, is suitable for estimating densities of *C.* *melanoneura*. Nevertheless, above a certain temperature threshold, prediction of *C.* *melanoneura* density might be skewed. No evidence was found that other relatively abundant psyllid species in the orchard, viz. *Baeopelma colorata*, *Cacopsylla breviantennata*, *Cacopsylla brunneipennis*, *Cacopsylla pruni* and *Trioza urticae*, were involved in ‘*Candidatus* Phytoplasma mali’ transmission. The results of our study contribute to an advanced understanding of insect vector behavior and thus have a practical impact for an improved field monitoring.

## 1. Introduction

Monitoring schemes for herbivore insects strongly depend on detailed knowledge about their specific requirements for natural resources, including food, mates or oviposition sites [1]. For arboricolous taxa in particular, knowledge about their distribution on different parts of a tree is also considered important to optimize sampling strategies for a meaningful density estimation [2].

The psyllids *Cacopsylla melanoneura* (Foerster, 1848) and *C. picta* (Foerster, 1848) (Hemiptera: Psylloidea: Psyllidae) are phloem feeding insects native to the Palaearctic region and associated with cultivated apple (*Malus* × *domestica*). While *C. melanoneura* is oligophagous on hawthorn (*Crataegus* spp.), apple (*Malus* spp.), medlar (*Mespilus germanica*) and pear (*Pyrus communis*), the only host plant of *C. picta* is apple [3,4]. Both species are univoltine and, similar to a number of other taxa of Psylloidea in the north temperate zone, they alternate between host and shelter plants during their life cycles [5,6]. Conifers serve as shelter plants for overwintering adults [7,8], while during the spring and summer *C. melanoneura* and *C. picta* spend four to five months on apple trees where they reproduce and where their immature stages develop [9,10,11]. In these months, adults as well as nymphs can harm the apple trees not just by their actual feeding activity, but mainly by transmitting the bacterium ‘*Candidatus* Phytoplasma mali’ [12,13,14,15]. This pathogen is the causal agent of apple proliferation (AP) [16], an economically important disease in commercial apple cultivation in Europe. Formation of witches’ brooms and enlarged, dentate stipules are specific symptoms that unambiguously characterize this disease [17,18]. However, the non-specific symptom of small, tasteless and colorless fruits is of economical relevance, since these fruits are not marketable [18]. The resulting yield losses in Italy for example have led to an financial damage of about 100 million Euro in 2001 [19]. Therefore, AP is a constant threat in apple growing regions. In the plant, ‘*Ca.* P. mali’ is residing in the phloem and can be acquired by psyllids during feeding on AP-infected trees. In the body of a competent vector, the bacterium is able to replicate and migrate to the salivary glands. Due to saliva release during the psyllid feeding activity, the pathogen can be transmitted to new plants [20].

In northern Italy, the overwintered adults (=remigrants) of *C. melanoneura* leave their shelter plants at the end of January or in February and fly to apple orchards. The population peaks in the second half of March and can be detected until April [10,11,21] or until full bloom [11]. In the beginning of March (when apple buds break), *C. melanoneura* starts to lay eggs and oviposition continues until mid-April. The first nymphs hatch in April and they can be found until May [21]. Adults of the new generation (=emigrants) of *C. melanoneura* occur in apple orchards from the end of April until mid-June [10,11,21] or during bloom until fruit development [11]. After this period on the apple tree, the adults migrate to conifers. The life cycle of *C. picta* is similar but staggered for approximately two months [10,11]. The peak of the *C. picta* remigrant population coincides with the bloom and emigrants of *C. picta* appear with starting fruit development [11].

*C. melanoneura* and *C. picta* are the only known competent vectors of ‘*Ca*. P. mali’ [14] and usually they are also the most abundant psyllid species in apple orchards. However, adults of several other species of Psylloidea developing on other host plants can be also collected on apple in lower numbers. Some of these species have been tested positive for the pathogen, but their role in its transmission has not been ascertained yet [22,23,24].

The monitoring of adult psyllid densities on fruit trees is usually carried out with beating trays or yellow sticky traps (e.g., [24,25,26,27]). Data generated with yellow sticky traps are cumulative and reflect the flight activity of the psyllids, while the beating tray method provides “snapshot” data on their presence at a given time and a defined location. Beating tray catches are known to be influenced by weather conditions, such as temperature [28,29], but the effect of temperature on the number of psyllid specimens collected has not been studied in detail yet. However, as psyllids are considered ectotherms [30], it can be assumed that temperature variations during the day might affect the capture success. Temperature can influence the behavior and biology of psyllids in various ways, e.g., in accelerating or extending the development of immature stages, as shown for *C. pyri* (Linnaeus, 1758) [31]. Temperature is also used as a driving variable to build forecasting models, e.g., to predict the beginning of the progressive arrival of *C. melanoneura* from their overwintering sites into apple orchards, based on a correlation between immigration dynamics and temperature [21]. A similar model has been developed for *C. melanoneura* and *C. picta* [32]. However, these models need further evaluation.

Some studies have reported a preference of certain psyllid species to colonize upper parts of the tree canopy. This has been documented for *Agonoscena targionii* (Lichtenstein, 1874) on pistachios [33], as well as *C. pyri* and *C. pyricola* (Foerster, 1848) on pear [29,34]. It is worth noting that higher numbers of sexually mature psyllid adults, eggs and nymphs were found in the upper canopy of pear trees [29,34]. However, the distribution patterns of *C. pyri* within a tree changed in the course of the vegetation period depending on the population density and the availability of suitable oviposition sites [34]. Distribution patterns of psyllids are not only influenced by endogenous but also by exogenous factors. Weather events can displace *C. pyricola* from a tree, but dislodged psyllids normally recolonize the tree after a short time [35]. Stratopoulou and Kapatos also found that *C. pyri* prefers to populate the southern and western sides of pear trees, which may be due to higher light intensities and temperatures [34]. Attraction to light has been demonstrated experimentally for some species of Psylloidea, e.g., *Diaphorina citri* (Kuwayama, 1908) [36] and the carrot psyllid, *Trioza apicalis* (Foerster, 1848) [37]. Under laboratory conditions, carrot psyllids preferred carrot leaves placed at higher light intensities [37]. Under field conditions, trap catches of the carrot psyllids and sunlight hours correlated stronger in autumn (when air temperatures were low) than in summer, indicating an interaction effect between temperature and light intensity [38].

Due to pragmatic reasons, AP vector monitoring with beating trays is commonly performed in the lower canopy (ca. 1–1.5 m above the ground) and throughout the entire day. Despite the high economic impact of *C. melanoneura* and *C. picta*, little is known about the small-scale spatio-diurnal dynamics of the AP-vector insects on apple trees. Thus, the following questions were addressed in the present study: (i) does the daytime (and the associated temperature) influence the number of AP vector catches? and (ii) is there any difference in the distribution of AP vectors at different canopy heights of apple trees? Based on the previously mentioned information on psyllid biology and behavior, we predicted to catch significantly more specimens of AP vectors in the morning and in the upper canopy. Furthermore, to analyze if other psyllid taxa were potentially involved in ‘*Ca*. P. mali’ transmission, quantitative PCR was performed to detect this pathogen in individuals of different Psylloidea species abundantly found in the orchard under study.

## 2. Results

### 2.1. Temperatures in the Apple Orchard

**Figure 1:**
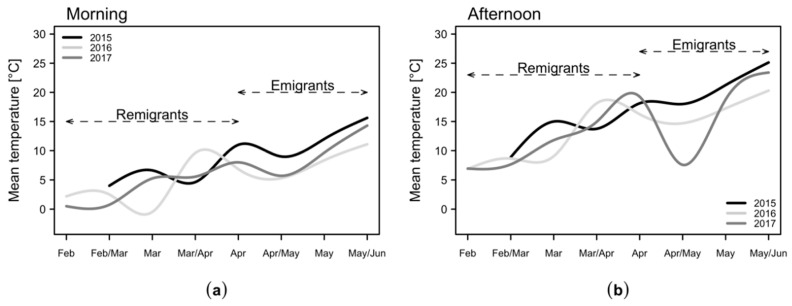
Mean temperatures (**a**) in the morning (7–9 a.m.) and (**b**) in the afternoon (2–4 p.m.) in the orchard from February to June 2015–2017. The presence of *C. melanoneura* remigrants and emigrants in the orchard is indicated by a dashed arrow-line.

Complete data on temperatures in the morning and afternoon are shown for each two calendar weeks (cw) in Appendix A Table A1. The temperatures varied in the three-year sampling period. In particular, the average temperatures in the morning and in the afternoon were higher from April to June 2015 compared to the subsequent two years (Figure 1). This time frame corresponds to the presence of *C. melanoneura* emigrants.

### 2.2. Psyllid Community

In total, 1021 specimens belonging to 28 species of Psylloidea were collected in the orchard during the three seasons of the study (Table 1). *C. melanoneura* was the most abundant species with a total of 630 specimens. *C. picta* was only found sporadically with a total of 13 specimens. Other Psylloidea species that were at least as abundant in the orchard as *C. picta* but do not reproduce on apple, were *Baeopelma colorata* (Löw, 1888), *C. pruni* (Scopoli, 1763), *C. brunneipennis* (Edwards, 1896), *Trioza urticae* (Linnaeus, 1758) and *C. breviantennata* (Flor, 1861).

### 2.3. Daytime Variation in the Numbers of C. melanoneura Specimens

**Figure 2:**
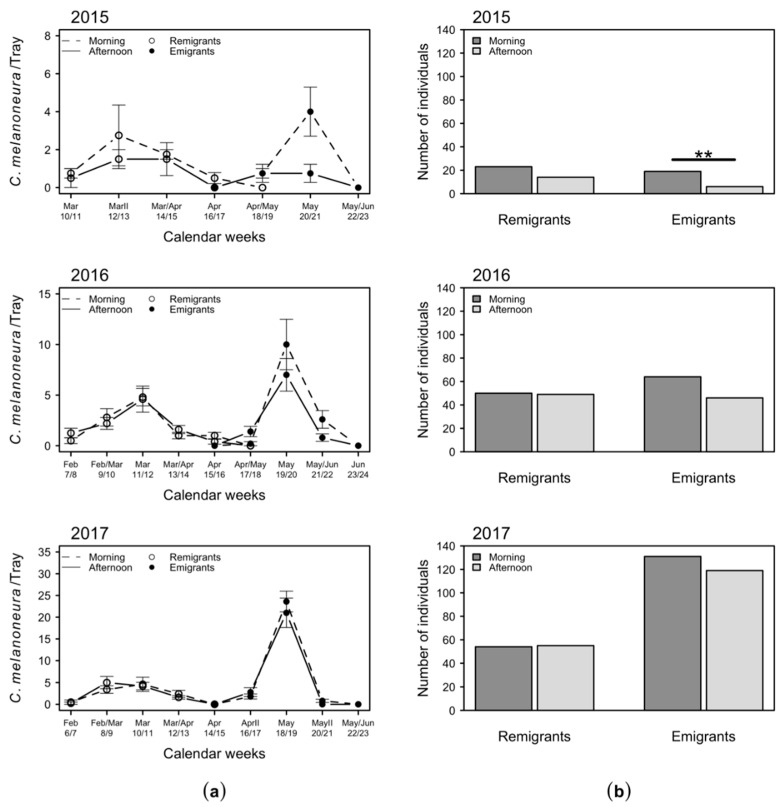
Numbers of *C. melanoneura* remigrant and emigrant specimens collected in the morning (7–9 a.m.) and in the afternoon (2–4 p.m.): (**a**) mean + SEM number of specimens collected in a bi-weekly routine per beating tray; (**b**) total numbers of specimens per year. Significant differences between groups are indicated with ** (*p* < 0.01).

Remigrants of *C. melanoneura*, which are recognizable by their prevailingly dark brown body, were detected from the beginning of March to the middle of April in 2015, from the middle of February to mid-April in 2016, and from mid-February until the end of March/beginning of April in 2017. In all three years, the light-green colored *C. melanoneura* emigrants were collected on apple trees from the end of April until the middle/end of May (Figure 2).

In March and May 2015, more *C. melanoneura* individuals were sampled in the morning (Figure 2a). Considering the total numbers of catches throughout the year, significantly more emigrants were collected in the morning than in the afternoon in 2015 (Figure 2b). In 2016 and 2017, *C. melanoneura* catches were similar in the morning and afternoon throughout the whole sampling period (Figure 2a) and only a slight trend of catching more emigrants in the morning was observed in these two years (Figure 2b). However, these differences were not statistically significant.

**Figure 3:**
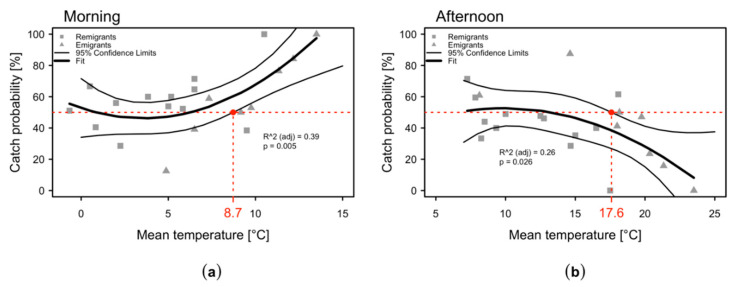
Probability of catching *C. melanoneura* specimens depending on the temperature (**a**) in the morning (7–9 a.m.); (**b**) in the afternoon (2–4 p.m.). Analysis was conducted to establish whether the probability of catching more *C. melanoneura* individuals is dependent on the temperature in the morning or in the afternoon. The 50% threshold (*y*-axis) indicates equal catch probabilities between morning and afternoon. If the temperature was above 8.7° C in the morning, the catch probability was higher (>50%) in the morning (**a**). If the temperature in the afternoon was below 17.6 °C, the catch probability was similar in the morning and in the afternoon, respectively (**b**).

**Figure A1:**
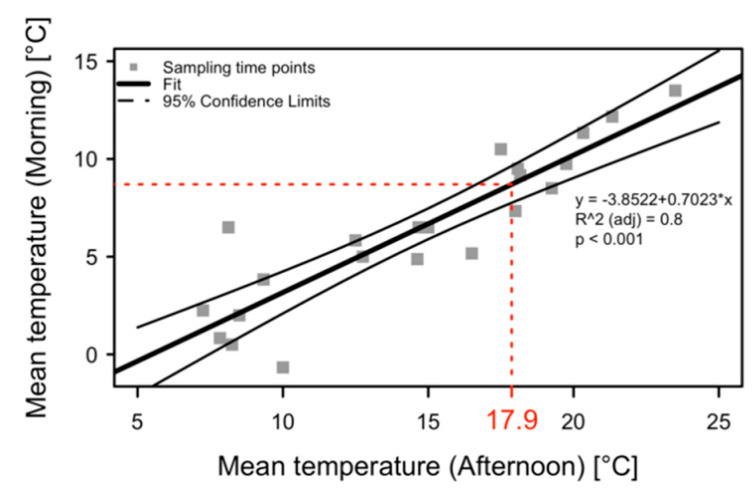
Correlation between mean temperature in the morning (7–9 a.m.) and mean temperature in the afternoon (2–4 p.m.) in the studied apple orchard in 2015–2017.

The probabilities to catch the same numbers of specimens in the morning as in the afternoon were similar when temperatures were below 8.7 °C in the morning or below 17.6 °C in the afternoon, respectively (Figure 3a,b). When temperatures exceeded these thresholds, more specimens were caught in the morning and less in the afternoon. Temperatures in the morning correlated positively with temperatures in the afternoon (Figure A1), i.e., if the temperatures exceeded 8.7° C in the morning they were above 17.6° C in the afternoon.

### 2.4. Variation of C. melanoneura Densities at Different Canopy Heights

**Figure 4:**
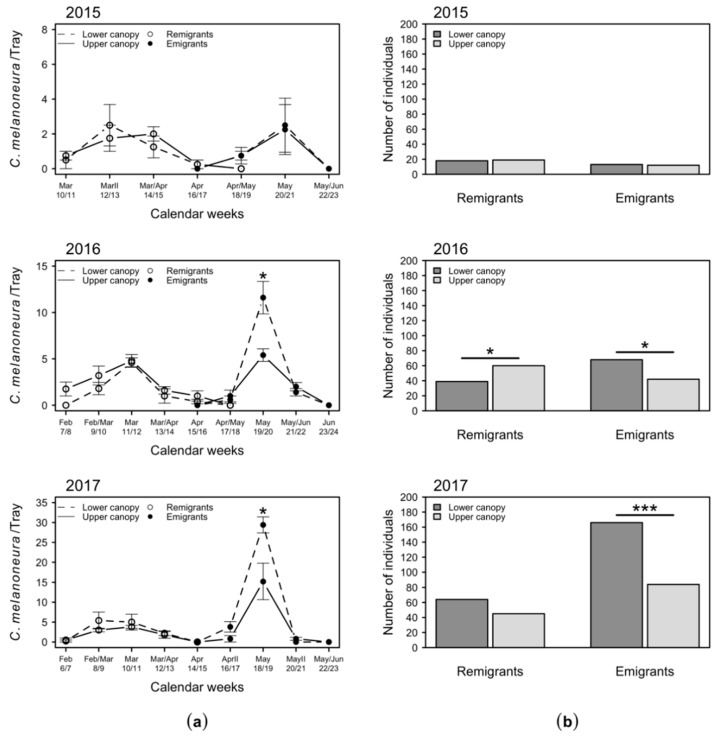
Numbers of *C. melanoneura* remigrant and emigrant specimens collected at the lower (1 m) and upper (2.5 m) canopy: (**a**) mean + SEM number of specimens collected in a bi-weekly routine per beating tray; (**b**) total catches per year. Significant differences between groups are indicated with * (*p* < 0.05) and *** (*p* < 0.001).

The sampling on apple trees was carried out at two different canopy heights, i.e., at 1 m and 2.5 m above the ground. In 2015, *C. melanoneura* catches from the two heights did not differ over the season (Figure 4a) and the totals of *C. melanoneura* remigrant and emigrant specimens were almost equal (Figure 4b). In 2016, similar numbers of *C. melanoneura* specimens were collected at the upper and lower canopy, except for May when the number of emigrants peaked and significantly more specimens were found in the lower canopy (Figure 4a). Furthermore, the total number of *C. melanoneura* remigrants was significantly higher in 2016 in the upper canopy, while significantly more *C. melanoneura* emigrants were collected in the lower canopy (Figure 4b). In 2017, the scenario was similar to 2016: i.e., in May significantly more individuals were caught in the lower canopy, while throughout the rest of the season between February and June, no differences were observed (Figure 4a). Over the whole season in 2017, significantly more emigrants were also found in the lower canopy (Figure 4b). The numbers of remigrant specimens did not differ between the two canopy heights in 2017 (Figure 4b).

**Figure 5:**
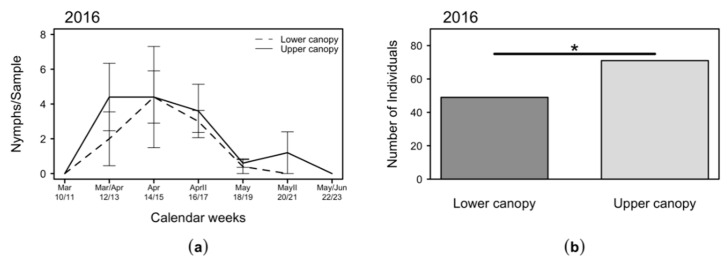
Numbers of *C. melanoneura* nymphs collected at the lower (1 m) and upper (2.5 m) canopy in 2016: (**a**) mean + SEM number of specimens collected in a bi-weekly routine per beating tray; (**b**) total catches per year. Significant differences between groups are indicated with * (*p* < 0.05).

The distribution of *C. melanoneura* nymphs at different canopy heights was analyzed in a single year (2016) (Figure 5). The nymphs occurred from the end of March/beginning of April, around six weeks after the arrival of the *C. melanoneura* remigrants and could be detected until mid-May, when *C. melanoneura* emigrant population peaked. During this period, similar numbers of nymphs were collected at the upper and lower canopy (Figure 5a), although the total number of nymphs per year was significantly higher in the upper canopy (Figure 5b).

### 2.5. Sampling of C. picta

Altogether, only 13 individuals of *C. picta* were collected in the three years of the study. This low number did not allow any statistical evaluation of the effects of daytime and canopy height on sampling. In 2015 and 2016, *C. picta* remigrants were recorded from April until the beginning of May. One *C. picta* emigrant was collected in the beginning of June 2015 and one in the beginning of July 2016, respectively. In 2017, no *C. picta* specimens were found.

### 2.6. Infection Rate with ‘Ca. P. mali’ in Selected Psylloidea Species

All species that were at least as abundant as *C. picta* in the orchards were subjected to a PCR analysis to detect if they carry AP-phytoplasma. In 2015, two out of nine *C. picta* specimens and one out of 62 *C. melanoneura* specimens were found infected with ‘*Ca*. P. mali’. In 2016 and 2017, two out of 208 and four out of 77 *C. melanoneura* specimens, respectively, were tested positive for ‘*Ca*. P. mali’. In 2015 and 2016, ‘*Ca*. P. mali’ was not detected in any other species of Psylloidea that were tested (Table 2). Altogether, seven AP-infected individuals of *C. melanoneura* and two AP-infected individuals of *C. picta* were collected in the three years of the study. This low number did not allow any statistical evaluation of the effects of daytime and canopy height on sampling AP-infected specimens.

## 3. Discussion

A reliable monitoring method is one of the most important prerequisites to estimate the actual density of an insect at a given location. An accurate estimation of these densities is particularly important if these insects transmit plant pathogens like phytoplasmas. The aim of this study was to evaluate if monitoring via the beating tray method (and how it is commonly performed) is a reliable approach for the estimation of the AP phytoplasma transmitting psyllids *C. picta* and *C. melanoneura*. In case of AP vectors, this sampling method is usually performed at a canopy height between 1 and 1.5 m and at arbitrary times of the day. However, it has been unclear if AP transmitting psyllids are more abundant in higher parts of the canopy and if more individuals can be collected in the morning when the insects are supposed to be less active. A bias due to a specific distribution of psyllids on trees and due to differences in their activity depending on daytime or temperature could affect the overall density estimation of the vectors. To address these principal questions, *C. melanoneura* and *C. picta* densities were monitored via beating tray sampling at two different daytimes and at two different canopy heights. A regression analysis was performed to analyze the correlation between temperature and the probability to catch more *C. melanoneura*. Based on this regression, a temperature threshold was determined. Two sampling time slots were chosen for the monitoring; a time slot from 7 until 9 a.m. when temperatures are generally lower and a time slot from 2 until 4 p.m. with higher temperatures. Further, two parts on the apple trees were distinguished—the lower and the upper canopy. Samples were taken at 1 m above the ground, where the lowest branches start to grow, and at 2.5 m, which represents the tops of the trees in the orchard under study. During the three-year survey, *C. picta* densities were too low to perform any statistical analysis. Therefore, we focused on the other AP vector, *C. melanoneura.* The following questions were addressed:

(i) Does the daytime have any effect on the beating tray sampling of *C. melanoneura*? In 2015, the number of emigrant specimens of *C. melanoneura* collected was significantly higher in the morning than in the afternoon. However, in the following two years, there was no significant difference and only a slight trend of more specimens in the morning was observed (Figure 2). In 2015, the mean temperatures from April to May/June were 2.5 °C to 4 °C higher in the morning and 3.4 °C to 3.5 °C higher in the afternoon as compared with 2016 and 2017 (Figure 1 and Table A1). Psyllids are considered ectotherms and thus sensitive to temperature fluctuations during the day and throughout the year [30]. Typically, ectotherms exhibit low activity at cold temperatures. Activity rises to an optimum but drops if temperatures increase further [39,40]. Therefore, it is generally recommended to perform beating in the morning, when temperatures are low and insects are inactive [28]. The higher temperatures in the afternoon in 2015 might have led to a higher jumping, flight and reaction activity of *C. melanoneura* and a reduced number of catches compared to the morning. In the relatively colder following years, the psyllids might have been generally less active in the afternoon due to lower temperatures and the difference between morning and afternoon catches was therefore less pronounced. The results indicate that *C. melanoneura* reacts to higher temperatures with an increased flight or jumping activity, which is reflected by less beating tray catches. This is consistent with the determined temperature thresholds of 8.7 °C in the morning or 17.6 °C in the afternoon, respectively (Figure 3). Since the temperatures in the morning and afternoon correlated positively, the threshold for the morning is an indicative value for the temperatures in the afternoon (Figure A1). The temperature threshold of 17.6 °C in the afternoon actually denotes that more specimens are likely to be collected in the morning (Figure 3). This implies that the flight activity of *C. melanoneura* at a temperature above 17.6 °C is increased. This temperature threshold was mainly reached from April onwards, when the emigrants started to appear. Indeed, the effect of catching more *C. melanoneura* individuals in the morning was only documented for emigrants (Figure 2). However, differences between catches in the morning and the afternoon were statistically significant only if temperatures were above 12 °C (in the morning) and 21.3 °C (in the afternoon) (Figure 3 and Table A1). Temperatures early in the season were below the threshold of 17.6 °C and therefore the general activity of remigrants is expected to be quite low throughout the whole day. This explains why no significant differences between the numbers of remigrants sampled in the morning and in the afternoon occurred.

(ii) Is there any difference in the distribution of *C. melanoneura* specimens at different canopy heights of apple trees? For some Psylloidea species, such as *C. pyri*, *C. pyricola* and *Agonoscena targionii*, it was shown that their adults prefer to dwell in the upper canopy [29,33,34]. The results of our study indicate that *C. melanoneura* also shows a non-random distribution on trees. For the remigrants, no difference was observed, but emigrants were collected in higher numbers from the lower canopy (Figure 4). To cross-evaluate the results from the beating tray sampling, a second method (wash down) was applied to analyze the distribution of the remigrant population, i.e., by quantifying their offspring. The mobility of psyllid nymphs is limited [41] since they lack jumping and flying abilities. Sampling of psyllid nymphs is very laborious but less prone to errors with regards to potentially skewed results caused by an escape of the individuals during the sampling procedure. The distribution pattern of the nymphs on apple trees strongly resembled the adults of the parental remigrant generation in the respective year (Figure 4 and Figure 5). This shows that the data acquired with the beating tray method are not strongly influenced by an escape-driven translocation of adult *C. melanoneura* remigrants during the sampling. The distribution patterns of the nymphs and the respective adult emigrants were not related. *C. melanoneura* emigrants might prefer to dwell in the lower parts of the canopy to avoid excessive sunlight and temperatures on the tops of the trees. This is in line with the findings of Rygg, who showed that trap catches of the carrot psyllid *Trioza apicalis* correlate negatively with light intensity and temperature [38]. The light intensity within the canopy ramps with canopy height from ca. 5–40% of full sun at the bottom (depending on the orchard training system) to 100% at the top of the canopy [42] and temperature is reduced approximately by 2 °C with 30–60% of shading in apple orchards [43]. Due to higher light intensity, *C. melanoneura* in the upper canopy is also surrounded by a warmer microclimate than in the lower parts of the tree. The higher number of specimens collected from presumably colder lower parts of the trees might thus be caused by a reduced activity of the insects, as described in the previous paragraph. Further, the light intensity at the bottom of the canopy decreases by around 15 to 30% (depending on the orchard training system) after petal fall [42]. Petal fall is followed by leaf growth that leads to shading and temperature reduction in the lower parts of the tree [43]. This might be a further reason why remigrants—which are present before petal fall [11]—are not as affected by shading effects as emigrants. Additionally, the weather may influence the *C. melanoneura* distribution on the trees. Horton et al. suggested that weather events displaced *C. pyricola* from the trees and that dislodged psyllids were able to recolonize the tree [35]. It cannot be excluded that rain or wind also affected the psyllid distribution in our study.

The density of *C. picta* in the orchard was low throughout the whole sampling period, in total only 13 specimens of *C. picta* were collected. This is in line with generally low densities of *C. picta* recently reported for South Tyrol [11]. Although the population densities of *C. picta* in the orchards are usually low also in other parts of Europe (e.g., [27]), this species is considered to be the main vector of apple proliferation wherever it is present [14]. Until now, it was unclear if the applied monitoring strategies are adequate to reveal the actual densities. The results of our study indicate that only few specimens of *C. picta* are present even in higher parts of the apple tree canopy, which might have been neglected in previous studies and monitoring.

The infection rate with ‘*Ca.* P. mali’ varied between 1.5% and 5.2% for *C. melanoneura* and was 22.2% for *C. picta* (Table 2). These results are similar to reported infection rates from South Tyrol and fit into the range reported also from other European countries [11,14]. In contrast to other studies [22,23,24], ‘*Ca.* P. mali’ was not detected in any other species of Psylloidea that were abundantly present in the orchard, further confirming that *C. melanoneura* and *C. picta* are the most relevant vectors of the apple proliferation phytoplasma in South Tyrol.

So far, no treatment against the apple proliferation phytoplasma has been available which could be applied in orchards. Therefore, the spread of ‘*Ca*. P. mali’ can be controlled only by uprooting of the infected trees and by phytosanitary measures against the insect vectors. There is a tendency that more emigrants of *C. melanoneura* can be sampled in the morning than in the afternoon and it should be taken into account that, if 17.6 °C is exceeded, the risk of underestimating the *C. melanoneura* population density increases if sampling is only performed in the afternoon. It is thus recommended that emigrant sampling is preferentially performed in the morning. For catching remigrants, beating tray sampling can be performed in the morning or in the afternoon. Sampling from the higher canopy of apple trees does not lead to increased numbers of psyllid catches, underlining that the currently used way of beating from the lower parts of trees is sufficient for the determination of *C. melanoneura*. In conclusion, the results of our study underline that the beating tray method is suitable for estimating densities of *C. melanoneura*. Nevertheless, above a certain temperature threshold, *C. melanoneura* density prediction might be skewed.

## 4. Materials and Methods

### 4.1. Study Site

The study was conducted in a commercial apple orchard located in the north-east of Merano (South Tyrol, Northern Italy, 700 m a.s.l., 46°64’26’’ N, 11°19’38’’ E) with approximately 1900 apple trees (mainly ’Golden Delicious’). The trees were planted between 2003 and 2009 and were, on average, 3 m tall. The orchard was treated twice a year with the agent Azadirachtin against aphids, which was first applied during blossom (middle of April) and then 2–4 weeks later (beginning of May). Based on visual inspections of specific symptoms carried out in autumn, the ‘*Ca*. P. mali’ infection rate of the apple trees in the orchard was determined as 1.6–2.4%. During sampling days, temperature was measured 1 m above ground in the shadow at 7 a.m., 9 a.m., 2 p.m. and 4 p.m.

### 4.2. Insect Sampling

Beating tray sampling of adults of Psylloidea was carried out from February to July in 2015, 2016 and 2017 in a bi-weekly routine. One branch per apple tree was struck four times using a padded stick, while a rectangular beating tray was held beneath the branch to collect the dislodged psyllids [29]. The sampling was carried out at two daytimes: in the morning (7–9 a.m.) and in the afternoon (2–4 p.m.), as well as at two different canopy heights: in the lower canopy (at 1 m above the ground) and in the upper canopy (at 2.5 m). Every two calendar weeks 50 trees (2015) as well as 40 trees (2016, 2017) were randomly selected and sampled for each sampling daytime and canopy height. This procedure was repeated four times in 2015 and five times in 2016, 2017. A total of 400 trees were sampled per routine.

Wash down sampling of nymphs of Psylloidea was carried out from March to July in 2016 in a bi-weekly routine. Ten shoots (ca. 10 cm long) per apple tree were taken at two different canopy heights: the lower canopy (at 1 m above the ground) and in the upper canopy (at 2.5 m) from 20 randomly selected trees for each two calendar weeks. This procedure was repeated five times and a total of 200 trees were sampled per routine. A plastic bag was carefully put over the shoots and those were cut. The plastic bags were closed tightly. Shoots were soaked for twelve hours in soapsuds at 4 °C in a closed vessel. Afterwards, the shoots were swayed for three minutes in the soapsuds. The shoots and the plastic bag were rinsed with water, which was collected and introduced to the soapsuds [44]. The soapsuds were filtrated (40 μm mesh size), so that nymphs were concentrated on the filter. The filter content was scanned for nymphs using a binocular.

### 4.3. Material Identification

Identification of all adult specimens of Psylloidea was performed based on morphology using identification keys [3,45,46]. The nomenclature and classification in the paper follows Ouvrard [4]. Specimens were stored in 75% ethanol at −20 °C. Specimens that could not be undoubtedly identified by morphology, have been additionally determined with the restriction fragment length polymorphism (RFLP) method [47]. Briefly, PCR was performed in a final volume of 20 µL containing 1× Colorless GoTaq^®^ Reaction Buffer (Promega, Madison, WI, USA), 0.2 mM dNTPs, 0.7 mM of each primer (qPSY-WG-F: 5’-TCA CGG GCG GCA ATG-3’; qPSY-WG-R: 5’-CCC ACA GCA CAT CAG ATC ACA-3’), 0.02 U GoTaq^®^ DNA polymerase (Promega, Madison, WI, USA) and 2.5 µL genomic DNA (1:10 diluted) under the following conditions: 2 min of initial denaturation at 95 °C; 45 cycles of 95 °C for 30 s, 46 °C for 30 s, and 72 °C for 1 min, and 5 min of final elongation at 72 °C. Restriction was performed with 10 µL of the amplicon (post PCR mix) and 0.5 U TaqαI restriction enzyme in 1× CutSmart™ (Thermo Fisher Scientific, Waltham, MA, USA) buffer at 65 °C for 4 h. The digested amplicon was visualized on a 2% Metaphor™ agarose gel and the characteristic digestion pattern was used for *Cacopsylla* species discrimination [47].

All nymphs were counted and fifth instar nymphs were identified based on morphological characteristics [3].

### 4.4. Detection of ‘Ca. P. mali’ in Psyllids

For the detection of ‘*Ca*. P. mali’ (and to perform RFLP, see above), genomic DNA was extracted from psyllid specimens using the DNeasy Blood and Tissue Kit (Qiagen, Hilden, Germany) [48].

‘*Ca*. P. mali’ infection rates were determined for *C. melanoneura, C. picta*, *B. colorata*, *C. pruni*, *C. brunneipennis*, *T. urticae* and *C. breviantennata*. Specific primers (rpAP15f-mod: 5’-TGC TGA AGC TAA TTT GGC-3’; rpAP15r3 5’-CCC ATG AAT ATT AAC CTC CT-3’) were used to detect ‘*Ca*. P. mali’ DNA in the samples [49]. PCR was performed in a final volume of 10 µL containing 2 µL of purified genomic DNA, 5 µL of 2X SYBR FAST qPCR Kit Master Mix, 0.25 µL of each primer (10 µM) and 2.5 µL molecular grade water, under the following conditions: 20 s of initial denaturation at 95 °C, 35 cycles of 95 °C for 3 s and 60 °C for 30 s, and a melting curve ramp from 65 to 95 °C at increments of 0.5 °C every 5 s [48].

### 4.5. Statistical Analysis

To compare the numbers of *C. melanoneura* specimens between the morning vs. afternoon sampling (all counts on a tree), a Mann–Whitney U test was performed. In addition, total counts per year of *C. melanoneura* remigrants as well as *C. melanoneura* emigrants at the morning vs. afternoon were compared using a Chi-square test. To compare the numbers of adult *C. melanoneura* specimens and nymphs collected at the lower vs. upper canopy (independently of the sampling time of day), the same tests as previously explained were applied. For *C. picta,* no statistical analysis was performed due to its low number of specimens and low frequency in the samples. For each two calendar weeks, the percentage of specimens caught in the morning and afternoon was calculated (catch probability). Based on the catch probability combined with the corresponding air temperatures in the morning and afternoon, a polynomial regression (2nd degree) was computed. The confidence intervals were defined at a level of 0.95. A threshold was set at a catch probability of 50% and with the lower confidence limit (morning) and the upper confidence limit (afternoon). The mean temperatures in the morning were correlated with the mean temperatures in the afternoon and a linear regression was computed. The confidence intervals were defined at a level of 0.95. No statistical analysis was performed to analyze the effect of sampling daytime and canopy height on the number of AP-infected *C. melanoneura* and *C. picta* specimens*,* due to the low number of AP-infected specimens. All analyses were carried out in the R statistical software (v. 3.1.3).

## Figures and Tables

**Table 1:** A list of species of Psylloidea collected in the apple orchard near Merano, South Tyrol, Italy in 2015–2017; n = numbers of both sexes together, n female = numbers of females only. * indicates individuals that could not be unambiguously identified by morphological and molecular means; ** indicates individuals that could not be identified due to missing body parts.

		2015		2016		2017		Total	
Family	Species	n	n Female	n	n Female	n	n Female	n	[%]
Psyllidae	*Cacopsylla melanoneura*	62	37	209	125	359	207	630	61.7
	*Baeopelma colorata*	66	25	26	14	45	25	137	13.4
	*Cacopsylla pruni*	19	9	27	15	17	9	63	6.2
	*Cacopsylla brunneipennis*			30	26	23	22	53	5.2
	*Cacopsylla breviantennata*	8	5	4	2	5	2	17	1.7
	*Cacopsylla picta*	9	4	4	0	-	-	13	1.3
	*Cacopsylla crataegi*	1	1	5	1	1	0	7	0.7
	*Cacopsylla affinis*	1	1	2	0	3	1	6	0.6
	*Cacopsylla pulchra*			3	2	2	0	5	0.5
	*Baeopelma foersteri*	1	0	1	1	1	0	3	0.3
	*Cacopsylla pulchella*			1	1	1	1	2	0.2
	*Cacopsylla mali*			2	1			2	0.2
	*Cacopsylla* spp. *	15	12	2	2			17	1.7
	*Psylla alni*	1	0					1	0.1
	*Cacopsylla peregrina*			1	0			1	0.1
Triozidae	*Trioza urticae*	9	4	4	3	8	5	21	2.1
	*Trioza remota*	2	1	5	2	2	2	9	0.9
	*Trioza rhamni*					7	5	7	0.7
	*Lauritrioza alacris*	3	1					3	0.3
	*Bactericera nigricornis*			1	1	2	2	3	0.3
	*Bactericera curvatinervis*	2	1					2	0.2
	*Bactericera acutipennis*			1	1	1	1	2	0.2
	*Trioza anthrisci*	1	1					1	0.1
	*Trioza rotundata*			1	1			1	0.1
	*Trioza* spp. **					1		1	0.1
Aphalaridae	*Rhinocola aceris*	2	1	1	1	5	2	8	0.8
	*Aphalara polygoni*	2	0					2	0.2
Homotomidae	*Homotoma ficus*	2	0					2	0.2
Liviidae	*Camarotoscena speciosa*			1	0			1	0.1
	*Psyllopsis fraxinicola*	1	1					1	0.1
	Number of species	18		21		17		28	
	Number of specimens	207		331		483		1021	

**Table 2:** Infection rates for ‘*Ca.* P. mali’ in the most abundant psyllid species in the studied apple orchard in 2015–2017; apple proliferation (AP)+ = ‘*Ca*. P. mali’ positive, n = number of specimens.

	2015		2016		2017	
	AP+/Analysed/Sampled	AP+	AP+/Analysed/Sampled	AP+	AP+/Analysed/Sampled	AP+
Species	[n]	[%]	[n]	[%]	[n]	[%]
*C. melanoneura*	1/62/62	1.51	2/208/209	0.96	4/77/359	5.19
*C. picta*	2/9/9	22.22	0/4/4	0	0/0/0	0
*B. colorata*	0/66/66	0	0/26/26	0		
*C. brunneipennis*			0/27/30	0		
*C. pruni*	0/19/19	0	0/27/27	0		
*C. breviantennata*	0/8/8	0	0/4/4	0		
*T. urticae*	0/9/9	0	0/4/4	0		

## Appendix A

**Table A1:** Minimum, maximum and mean temperatures at two daytimes of sampling (morning: 7–9 a.m.; afternoon: 2–4 p.m.), in the studied apple orchard per each two calendar weeks from 2015 until 2017.

**Month 2015**		**Mar**	**MarII**	**Mar/Apr**	**Apr**	**Apr/May**	**May**	**May/Jun**
**Calendar Weeks 2015**		**10/11**	**12/13**	**14/15**	**16/17**	**18/19**	**20/21**	**22/23**
Min_morning_ [°C]		3	5	3.5	8	8.5	10.5	13.5
Max_morning_ [°C]		4.5	7.5	7	12.5	10.5	14.5	16.5
Mean_morning_ [°C]		4	6.6	4.6	11	9	12	15.6
Min_afternoon_ [°C]		7.5	13.5	9.5	12.5	16	21	22.5
Max_afternoon_ [°C]		11	16.5	16.5	20	21	22	28
Mean_afternoon_ [°C]		8.9	15	13.8	18.1	18	21.3	25.1
**Month 2016**	**Feb**	**Feb/Mar**	**Mar**	**Mar/Apr**	**Apr**	**Apr/May**	**May**	**May/Jun**
**Calendar Weeks 2016**	**7/8**	**9/10**	**11/12**	**13/14**	**15/16**	**17/18**	**19/20**	**21/22**
Min_morning_ [°C]	1.5	1	−2.3	8.5	4	−1	3.5	10
Max_morning_ [°C]	3.3	4	0.5	10.5	8.5	9	10.5	13
Mean_morning_ [°C]	2.2	2.5	−0.5	9.6	6.9	5.3	8.4	11.1
Min_afternoon_ [°C]	6.5	7.5	6	17	7.5	8	13.5	16.5
Max_afternoon_ [°C]	8	9.5	12.5	19.5	19	19	24	23
Mean_afternoon_ [°C]	6.9	8.7	9.1	18.1	16.3	14.7	17.3	20.3
**Month 2017**	**Feb**	**Feb/Mar**	**Mar**	**Mar/Apr**	**Apr**	**AprII**	**May**	**MayII**
**Calendar Weeks 2017**	**6/7**	**8/9**	**10/11**	**12/13**	**14/15**	**16/17**	**18/19**	**20/21**
Min_morning_ [°C]	0.5	−0.5	4	3	6.5	4.5	9.5	11.5
Max_morning_ [°C]	0.5	2	8	6.5	9.5	8.5	10	15.5
Mean_morning_ [°C]	0.5	0.8	5.3	5.6	8	5.7	9.7	14.3
Min_afternoon_ [°C]	6.5	6	8	9.5	18.5	6.8	16.5	23
Max_afternoon_ [°C]	10	11.5	19	22	20	9.5	23	24
Mean_afternoon_ [°C]	6.9	7.7	11.8	15	19.3	7.6	18.8	23.4
